# Unique and shared techniques in cognitive-behavioural and short-term psychodynamic psychotherapy: a content analysis of randomised trials on depression

**DOI:** 10.1080/21642850.2014.931231

**Published:** 2014-09-10

**Authors:** Jürgen Barth, Nadja Michlig, Thomas Munder

**Affiliations:** ^a^ Institute of Social and Preventive Medicine, University of Bern, Bern, Switzerland; ^b^ Institute for Complementary and Integrative Medicine, University of Zürich, Zürich, Switzerland

**Keywords:** cognitive-behavioural therapy, short-term psychodynamic psychotherapy, psychotherapeutic techniques, depression, randomised trials, meta-analysis

## Abstract

Randomised controlled trials (RCTs) of psychotherapeutic interventions assume that specific techniques are used in treatments, which are responsible for changes in the client's symptoms. This assumption also holds true for meta-analyses, where evidence for specific interventions and techniques is compiled. However, it has also been argued that different treatments share important techniques and that an upcoming consensus about useful treatment strategies is leading to a greater integration of treatments. This makes assumptions about the effectiveness of specific interventions ingredients questionable if the shared (common) techniques are more often used in interventions than are the unique techniques. This study investigated the unique or shared techniques in RCTs of cognitive-behavioural therapy (CBT) and short-term psychodynamic psychotherapy (STPP). Psychotherapeutic techniques were coded from 42 masked treatment descriptions of RCTs in the field of depression (1979–2010). CBT techniques were often used in studies identified as either CBT or STPP. However, STPP techniques were only used in STPP-identified studies. Empirical clustering of treatment descriptions did not confirm the original distinction of CBT versus STPP, but instead showed substantial heterogeneity within both approaches. Extraction of psychotherapeutic techniques from the treatment descriptions is feasible and could be used as a content-based approach to classify treatments in systematic reviews and meta-analyses.

## Introduction

Cognitive-behavioural therapy (CBT) and short-term psychodynamic psychotherapy (STPP) are commonly regarded as two distinct psychotherapeutic treatments that differ in multiple aspects: mechanism of change, theoretical models, treatment rationales, and education modalities (Watzke, 2002; Watzke, Koch, & Schulz, 2006), as well as in their therapeutic techniques and strategies (Blagys & Hilsenroth, 2000; Jones & Pulos, 1993; Leichsenring, Hiller, Weissberg, & Leibing, 2006; Watzke et al., 2006). However, some authors argue that in the last years a consensus regarding the most useful treatment strategies for psychotherapeutic interventions was achieved (Beitman, Goldfried, & Norcross, 1989; Goldfried, 2009). Therefore, the integration of strategies and techniques from one type of psychotherapy to another is already happening (Goldfried, 2009). Examples include the integration of exposure in STPP for social phobia (Leichsenring et al., 2006), the emphasis of childhood experiences in CBT (Young, 1994), or the integration of STPP and Interpersonal Therapy into CBT in the Cognitive Behavioural Analysis System of Psychotherapy (Keller et al., 2000).

Blagys and Hilsenroth (2002) examined psychotherapy process studies and identified six unique techniques of CBT. This review focused on therapist activities and interventions that differentiated CBT from STPP in at least two different studies and at least two different research sites. The six exclusive CBT techniques are as follows, with the level of empirical support given in parentheses: (1) homework assignments (strong support); (2) direction of the session's activities (strong support); (3) teaching skills to patients (strong support); (4) emphasis on future experiences (strong support); (5) providing patients with information about their treatment, diagnoses or symptoms (moderate support); and (6) a cognitive/intrapersonal focus (moderate support).

In an earlier study with the same approach, seven techniques that are exclusively used in STPP were identified (Blagys & Hilsenroth, 2000). The techniques are as follows: (1) focus on affect and the expression of the patient's emotions (strong support); (2) explore the patient's attempts to avoid topics or engage in activities that hinder the progress of therapy (strong support); (3) identify patterns in the patient's actions, thoughts, feelings, experiences and relationships (strong support); (4) place an emphasis on past experiences (strong support); (5) focus on the patient's interpersonal experiences (strong support); (6) place an emphasis on the therapeutic relationship (moderate support); and (7) explore the patient's wishes, dreams or fantasies (moderate support).

Other studies focused on the shared features of CBT and STPP. In terms of psychotherapy outcome research, Garfield (1996) summarised the common aspects, such as a good therapeutic relationship, the creation of hope, an improvement of coping skills and a provision of an explanation for the client's problems. According to Castonguay, Goldfried, Wiser, Raue, and Hayes (1996), a therapeutic alliance and the process of client experiencing emotions are common in many psychotherapeutic approaches. Other authors, such as Grencavage and Norcross (1990), came to similar conclusions about commonly used techniques: the development of a therapeutic alliance, opportunities for catharsis, the acquisition and practice of new behaviours, and the client's positive expectancies.

Furthermore, there is also a debate regarding the homogeneity of the strategies and techniques within the CBT and STPP treatment approaches (Leichsenring et al., 2006; Linden & Hautzinger, 2008; Watzke, 2002; Watzke et al., 2006). Leichsenring et al. (2006) assumed the existence of a supportive-expressive continuum of psychodynamic interventions, with a broad variety of techniques. Several different treatments are summarised as STPP, such as short psychodynamic supportive psychotherapy (according to De Jonghe, Kool, Van Aalst, Dekker, & Peen, 2001; Dekker et al., 2008) and insight-oriented psychotherapy (O'Leary & Cradock O'Leary, 2011). In addition, it is well known that many different treatments are united under the label ‘CBT’ (Linden & Hautzinger, 2008; Watzke, 2002), such as interpersonal therapy, self-system therapy, and acceptance and commitment therapy (APA, 2006).

This study investigates the homogeneity and heterogeneity of therapeutic techniques within and between CBT and STPP in randomised trials (RCTs). RCTs are considered to implement clearly defined homogenous treatments, as compared to treatments in clinical practice, where substantial heterogeneity has been observed in therapists trained within the same treatment approach (Watzke, Rueddel, Koch, Rudolph, & Schulz, 2008). However, no studies on the unique and shared techniques of treatments in RCTs have been conducted.

In conclusion, there is an on-going debate about the unique and shared techniques in specific treatments. But, this debate is often driven by expert opinions and clinical observations. This study aims to empirically investigate whether or not treatment approaches that are labelled as CBT or STPP in randomised studies use the same psychotherapeutic techniques to treat depression, or if some of the psychotherapeutic techniques are used exclusively in one of the mentioned treatment approaches. In addition, we investigated whether or not the psychotherapeutic techniques can build up clusters of interventions, which correspond to the original labels of CBT and STPP. Finally, the change of psychotherapeutic techniques used in both treatment approaches over time is studied, and we investigated whether or not there is a consensus between the different treatment approaches coming up.

## Method

### Data source

Relevant studies were selected from a comprehensive database that included 281 randomised controlled trials of psychotherapy in the treatment of adult depression. In 2010, Cuijpers (Barth et al., 2013; Cuijpers, 2010, 2011) set up this database. In our review, we included studies that were (a) published between 1979 and 2010, (b) contained either a CBT or STPP treatment delivered in an individual face-to-face setting, (c) were published in either English or German, and (d) contained a treatment description.

### Study selection procedure

Of the 281 studies, 68 met the inclusion criteria. Of these, 14 studies contained an STPP treatment and 59 studies contained a CBT treatment (5 studies contained both CBT and STPP within 1 study). In order to compare studies that are similar in terms of their characteristics of the treatment description independent of the content, we matched two CBT treatments (*n* = 28) to each available STPP treatment (*n* = 14), according to the following pre-specified characteristics. The following matching criteria were used, listed in order of importance: (a) same study (treatments from the same study were matched; *n* = 5); (b) target group (e.g. adult depression, women with postpartum depression, elderly, other); (c) number of words in the treatment descriptions; and (d) publication year. The flow chart of study inclusion is presented in Appendix 1.

### Development of rating manual

The first goal of this study was to develop a rating manual that represents the psychotherapeutic techniques used in CBT and STPP treatment approaches. The selection of techniques for the manual took place in three steps. The first step was clarification of the conceptual background of the two treatment approaches. For this purpose, established psychotherapeutic techniques used in CBT and STPP were extracted from the literature (Blagys & Hilsenroth, 2000, 2002; Driessen et al., 2010; Kächele & Thomä, 2006; Kohut, Goldberg, & Stepansky, 1984; Leichsenring et al., 2006; Michie, Johnston, Francis, Hardeman, & Eccles, 2008; Pfammatter, Barth, Gerger, Munder, & Tschacher, 2010; Pfammatter & Tschacher, 2012; Trijsburg et al., 2002; Watzke et al., 2006).

A first draft of the rating manual was created in late 2010. This rating manual contained a section of cognitive-behavioural techniques, psychodynamic techniques, and general psychotherapeutic techniques. General psychotherapeutic techniques included those that were likely to be present in both approaches, but were not specific for either approach (e.g. empathy). For each technique, the manual contained a *label* (e.g. relaxation), a *definition* (e.g. techniques to reduce physiological arousal), and *synonyms* (e.g. autogenic training).

The second step involved the rating manual being externally validated by experts with theoretical knowledge and practical experience in CBT and/or STPP. These external experts were: Birgit Watzke (University of Hamburg-Eppendorf, Germany), Manfred E. Beutel (University of Mainz, Germany), and Tony Roth (University College of London, UK). The experts were asked to comment on the labels, definitions, and synonyms in the manual. The experts' suggestions, ideas, and corrections were used to revise the manual in early 2011.

Third, the manual was piloted and minor changes to the item definitions were made. The final manual was finished in March 2011 and contained 29 psychotherapeutic techniques (cognitive-behavioural techniques = 12; psychodynamic techniques = 9; and general psychotherapeutic techniques = 8). The final rating manual is included in Appendix 2.

### Rating procedure

Masked treatment descriptions from all studies were extracted from the research reports. Information about the study (e.g. author, publication year, and journal title) and the treatment approach were removed. The treatment descriptions were presented to the raters in a random order. The techniques were coded using the computerised data entry system, Epidata (Lauritsen, 2008). For each treatment description, all of the techniques were rated as being *present* (i.e. techniques have explicitly been used), *not present* (i.e. techniques have explicitly not been used), or *unclear* (i.e. no information regarding the presence or the absence of techniques was available). Two persons – a master's student in clinical psychology (N. M.) and a Ph.D.-level psychologist (J. B.) – independently rated all of the treatment descriptions after receiving extensive training. Disagreements were resolved by discussing the issues and coming to a consensus. The two raters and a neutral third party (a Ph.D. student [T. M.]) were involved in the discussions.

Inter-rater reliability was determined separately for each technique, using Cohen's *κ* (Fleiss, 1975). The *κ* can be interpreted by using cut-offs, as described by Cicchetti (1994): the rater agreement is low if *κ* < .40, fair if κ is between ≥.40 and <.60, good if *κ* is between ≥.60 and <.75, and excellent if *κ* ≥ .75. Overall, the median *κ* per group of psychotherapeutic techniques was excellent (*κ* = .81; cognitive-behavioural techniques: *κ* = .91, psychodynamic techniques: *κ* = .84, general psychotherapeutic techniques: *κ* = .79), with agreement for the individual techniques ranging from *κ* = .37 to *κ* = 1. Rater agreement was in the low range for one technique (self-instruction: *κ* = .37) and in the moderate range for three techniques (role-play: *κ* = .48, problem-solving technique: *κ* = .48, empathy: *κ* = .50). All of the other techniques achieved good and excellent inter-rater reliability (see Appendix 3).

### Statistical analysis

The differences in psychotherapeutic techniques used with CBT and STPP treatments were analysed to determine techniques that occurred with a frequency of at least 10% in either CBT or STPP treatments using *χ* ^2^-tests and for cells with less than five counts Fishers exact test. A *p*-value of ≤.05 was defined as a cut-off for exclusively used techniques. A *p*-value of ≥.30 was defined as a cut-off for techniques shared by both approaches. The somewhat arbitrary cut-off for shared techniques was chosen based on the rationale that *p*-values between .05 and .30 do not allow for a clear interpretation of whether techniques are unique or shared. The same procedure was used to investigate changes within the approaches over time. Studies published in 1995 or earlier were contrasted with studies published after 1996. A hierarchical cluster analysis was used to derive homogenous groups of treatments that were similar with regards to the techniques used (Borgen & Barnett, 1987).

The results of the cluster analysis were validated using a linear discriminant analysis (Bortz, 1999). In the linear discriminant analysis, the classification of the studies was set as valid and the appropriateness was checked by a reclassification according to the techniques. If the total number of categories matched the original classification, and if the variables could be distinguished between the clusters, the initial findings of the cluster analysis were confirmed.

In addition to this strategy, a hierarchical cluster analysis from three random samples taken from the original study sample was employed (80% of all studies). The classification of the reduced set of studies was compared to the original classification with all of the studies in order to test the accuracy of the classification. Cohen's *κ*, as a reliability coefficient (Fleiss, 1975), was computed from the originally defined cluster and the clusters from the three random samples. Two general psychotherapeutic techniques (inclusion of significant others and self-disclosure) were never rated by either of the two raters and thus were excluded from these analyses. Few techniques were coded as being *not present*. Consequently, the categories *not present* and *unclear* were collapsed. All analyses were carried out using SPSS 19 (Norusis, 2010).

## Results

### Study characteristics

We included 28 CBT studies and 14 STPP studies in our analysis. The included studies were published between 1979 and 2010 (*M* = 1996.93, SD = 10.91). The number of words in the treatment descriptions ranged from 17 to 618 (*M* = 157.88, SD = 146.45), and the number of described psychotherapeutic techniques in these treatment descriptions ranged from 1 to 13 (*M* = 5.52, SD = 3.39). The treatment length ranged from 6 to 30 sessions (*M* = 14.24, SD = 5.05). Descriptive information for the included studies is presented in Table 1, and in an earlier comprehensive review of all of the available studies on depression (Barth et al., 2013).

**Table 1.** Descriptive information of all included studies (*n* = 42).

Authors	Target group	Words	Techniques	Sessions	Treatments
		(*n*)	(*n*)	(*n*)	
1. Baker et al. (2010)	Others	238	10	10	CBT
2. Beach and O'Leary (1992)	Adults	79	2	18	CBT
3. Beck, Young, Bedrosian, and Budenz (1985)	Adults	37	2	20	CBT
4. Bellack, Hersen, and Himmelhoch (1981)	Adults	39	1	12	STPP
5. Beutler et al. (2003)	Others	46	2	20	CBT
6. Bodenmann et al. (2008)	Adults	70	3	20	CBT
7. Brown and Lewinsohn (1984)	Adults	505	11	12	CBT
8. Burnand, Andreoli, Kolatte, Venturini, and Rosset (2002)	Adults	164	13	10	STPP
9. Carrington (1979)	Adults	618	12	12	STPP
10. Carrington (1979)	Adults	523	5	12	CBT
11. Castonguay et al. (2004)	Adults	241	7	16	CBT
12. Cho, Kwon, and Lee (2008)	PPD	148	7	9	CBT
13. Conradi et al. (2007)	Adults	74	4	14	CBT
14. Coopers, Murray, Wilson, and Romaniuk (2003)	PPD	46	1	10	STPP
15. Coopers et al. (2003)	PPD	136	5	10	CBT
16. David, Szentagotai, Lupu, and Cosman (2008)	Adults	54	2	20	CBT
17. De Jonghe, Kool, Van Aalst, Dekker, and Peen (2001)	Adults	143	8	16	STPP
18. Dekker et al. (2008)	Adults	33	2	16	STPP
19. Dimidjian et al. (2006)	Adults	128	7	16	CBT
20. Dozois et al. (2009)	Adults	40	4	15	CBT
21. Fry (1984)	Elderly	596	12	12	CBT
22. Gallagher and Thompson (1982)	Elderly	145	6	16	STPP
23. Gallagher and Thompson (1982)	Elderly	200	4	16	CBT
24. Gallagher-Thompson and Steffen (1994)	Others	82	6	20	STPP
25. Gallagher-Thompson and Steffen (1994)	Others	41	2	20	CBT
26. Jacobson et al. (1996)	Adults	227	2	20	CBT
27. Kay-Lambkin, Baker, Lewin, and Carr (2009)	Others	17	2	10	CBT
28. Laidlaw et al. (2008)	Elderly	144	4	8	CBT
29. Macaskill and Macaskill (1996)	Adults	61	4	30	CBT
30. Maina, Forner, and Bogetto (2005)	Adults	203	12	20	STPP
31. Maina, Rosso, Rigardetto, Piat, and Bogetto (2010)	Others	264	10	13	STPP
32. McKnight, Nelson-Gray, and Barnhill (1992)	Adults	94	5	8	CBT
33. McLean and Hakstian (1979)	Adults	192	8	10	STPP
34. McNamara and Horan (1986)	Adults	126	3	9	CBT
35. Rush and Watkins (1981)	Adults	88	3	20	CBT
36. Salminen et al. (2008)	Adults	66	2	16	STPP
37. Schmidt and Miller (1983)	Adults	165	7	8	CBT
38. Scott, Tacchi, Jones, and Scott (1997)	Adults	107	8	6	CBT
39. Shamsaei, Rahimi, Zarabian, and Sedehi (2008)	Adults	121	9	8	CBT
40. Shapiro et al. (1994)	Adults	69	5	–	STPP
41. Shapiro et al. (1994)	Adults	55	3	–	CBT
42. Vitriol, Ballesteros, Florenzano, Weil, and Benadof (2009)	Others	206	7	12	STPP

Notes: *n* = number, CBT = cognitive-behavioural treatment approach, STPP = short-term psychodynamic treatment approach, PPD = post-partum depression, the target group ‘others’ summarise specific populations like comorbid depression and drug abuse or caregivers.

### Comparison of descriptions of CBT versus STPP treatment approaches

In order to verify the matching procedure, the descriptive information was compared and demonstrated satisfying results (see Table 2). Similarities were found in publication year (CBT: *M* = 1997.00, SD = 10.60; STPP: *M* = 1996.79, SD = 11.90; *p* = .967), number of sessions (CBT: *M* = 14.33, SD = 5.72; STPP: *M* = 14.05, SD = 3.46; *p* = .115), number of psychotherapeutic techniques in the treatment descriptions (CBT: *M* = 4.96, SD = 2.89; STPP: *M* = 6.64, SD = 3.99; *p* = .625), and number of words in the treatment descriptions (CBT: *M* = 155.75, SD = 147.28; STPP: *M* = 162.14, SD = 144.66; *p* = .183).

**Table 2.** Characteristics of CBT- and STPP-labelled treatment approaches.

	CBT treatments (*n* = 28)		STPP treatments (*n* = 14)		
Characteristics of CBT and STPP treatments	*M*	SD	*M*	SD	*p* (*χ*^2^)
1. Publication year	1997.00	10.60	1996.79	11.90	.967
2. Number of words in treatment descriptions	155.75	147.28	162.14	144.66	.183
3. Number of psychotherapeutic techniques	4.96	2.89	6.64	3.99	.625
4. Number of sessions	14.33	5.72	14.05	3.46	.115

Notes: *M* = mean, SD = standard deviation, *p* (*χ* ^2^) = chi-square test.

### Shared techniques in CBT and STPP

The psychotherapeutic techniques shared by CBT and STPP are presented in Table 3. In addition to ‘focus on strengths’ (*p* = .590) as a general psychotherapeutic technique, only techniques from the CBT section were shared by both treatment approaches. These included: structuring of treatment/session (*p* = .656), homework assignments (*p* = .485), education about the disorder (*p* = .485), reinforcement (*p* = 1.000), and setting treatment goals (*p* = 1.000).

**Table 3.** Shared psychotherapeutic techniques of CBT- and STPP-labelled treatment approaches (*p* ≥ .30).

Psychotherapeutic techniques according to approaches		CBT treatments (*n* = 28)		STPP treatments (*n* = 14)		
	* *	*n*	%	*n*	%	*p (χ^2^)*
CBT	Structuring of treatment/session	12	42.85	5	35.71	.656
	Homework assignments	10	35.71	3	21.42	.485
	Education about the disorder	10	35.71	3	21.42	.485
	Reinforcement	5	17.85	3	21.42	1.000
	Setting treatment goals	5	17.85	2	14.28	1.000
PDT	None					
General	Focus on strength	2	7.14	2	14.28	.590

Notes: *n* = number of used techniques, % = percentage of used techniques, *p* (*χ* ^2^) = chi-square test, CBT = cognitive-behavioural techniques, PDT = psychodynamic techniques, and general = general psychotherapeutic techniques.

### Exclusively used techniques in CBT and STPP

The psychotherapeutic techniques exclusively used in either CBT or STPP treatments are shown in Table 4. STPP utilises a large number of exclusive psychodynamic techniques. These techniques included: emphasis on past experiences (*p* < .001), focus on the therapeutic relationship (*p* < .001), interpretation (*p* < .001), working with unpleasant emotions (*p* < .001), transference (*p* < .001), working with unconscious conflicts (*p* < .002), therapeutic abstinence (*p* = .032), and resistance-analysis (*p* = .032).

**Table 4.** Exclusively used psychotherapeutic techniques of CBT- and STPP-labelled treatment approaches (*p* ≤ .05).

		CBT treatments (*n* = 28)		STPP treatments (*n* = 14)		
Psychotherapeutic techniques according to approaches		*n*	%	*n*	%	*p* (*χ*^2^)
CBT	Working with dysfunctional cognitions	25	89.28	3	21.42	.001
	Monitoring	13	46.42	1	7.14	.015
	Activation	12	42.42	1	7.14	.031
PDT	Emphasis on past experiences	3	10.71	9	64.28	.001
	Focus on therapeutic relationship	1	3.57	8	57.14	.001
	Interpretation	0	0	9	64.28	.001
	Working with unpleasant emotions	0	0	8	57.14	.001
	Transference	0	0	7	50.00	.001
	Working with unconscious conflicts	0	0	5	35.71	.002
	Resistance-analysis	0	0	3	21.42	.032
	Therapeutic abstinence/neutrality	0	0	3	21.42	.032
General	Skills training	11	39.28	0	0	.007
	Advice/support	4	14.28	6	42.85	.059
	Emotional activation	0	0	5	35.71	.002

Notes: *n* = number of used techniques, % = percentage of used techniques, *p* (*χ* ^2^) = chi-square test, CBT = cognitive-behavioural techniques, PDT = psychodynamic techniques, and general = general psychotherapeutic techniques.

The most frequently used cognitive-behavioural techniques in CBT treatments included: working with dysfunctional cognitions (*p* < .001), monitoring (*p* = .015), and activation (*p* = .031). Skills training (*p* = .007), as a general psychotherapeutic technique, was uniquely used in CBT treatments (39.2%). Advice/support (*p* = .059) and emotional activation (*p* < .002) were more often used in STPP than in CBT treatments (advice/support: CBT = 14.2%, STPP = 42.8%; emotional activation: CBT = 0%, STPP = 35.7%).

### Empirical clustering of studies according to psychotherapeutic techniques

**Figure 1.**
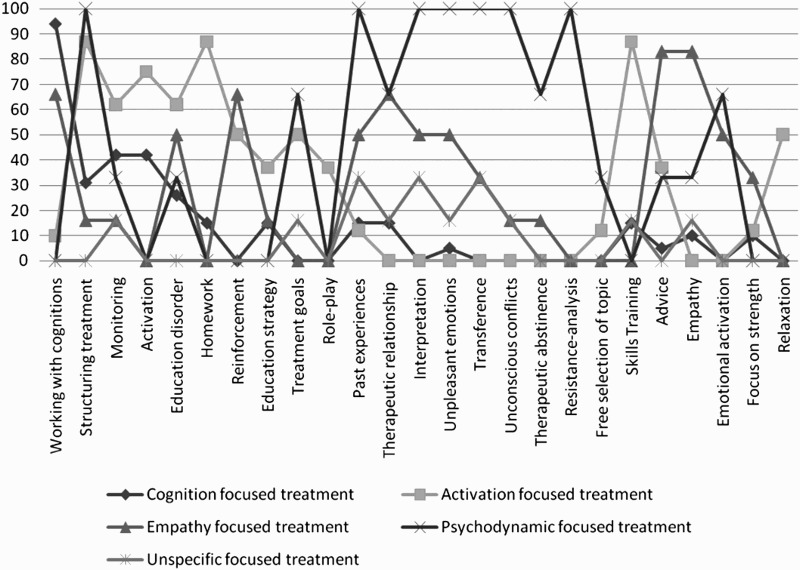
Graph of the described psychotherapeutic techniques in the five clusters (prevalence in % in each cluster).

A cluster analysis of the techniques resulted in five treatments clusters. The decision for a five-cluster solution was driven by the goal of providing valid treatment groups. A four-cluster solution was not considered because 59.5% of all of the treatments fit within a single cluster. A six-cluster solution was not chosen because two clusters were very similar. The five clusters were labelled as follows: (1) cognition-focused treatment (*n* = 19); (2) activation-focused treatment (*n* = 8); (3) empathy-focused treatment (*n* = 6); (4) unspecific-focused treatment (*n* = 6), and (5) psychodynamic-focused treatment (*n* = 3). Table 5 and Figure 1 show techniques associated with each of the clusters, as well as the percentage of their use within each of the five clusters.

**Table 5.** Used psychotherapeutic techniques in the five clusters.

		Cognition-focused treatment (*n* = 19)		Activation-focused treatment (*n* = 8)		Empathy-focused treatment (*n* = 6)		Unspecific-focused treatment (*n* = 6)		Psychodynamic-focused treatment (*n* = 3)		Total
		*n*	%	*n*	%	*n*	%	*n*	%	*n*	%	*n*
*Psychotherapeutic techniques*												
CBT	Working with dysfunctional cognitions	18	94.73	8	100	4	66.67	0	0	0	0	30
	Structuring of treatment/session	6	31.57	7	87.50	1	16.67	0	0	3	100	17
	Monitoring	8	42.10	5	62.50	1	16.67	1	16.67	1	33.33	16
	Activation	8	42.10	6	75.00	0	0	0	0	0	0	14
	Education about the disorder	5	26.31	5	62.50	3	50.00	0	0	1	33.33	14
	Homework assignments	3	15.78	7	87.50	0	0	0	0	0	0	10
	Reinforcement	0	0	4	50.00	4	66.66	0	0	0	0	8
	Education about the therapeutic strategy	3	15.78	3	37.50	1	16.67	0	0	0	0	7
	Setting treatment goals	0	0	4	50.00	0	0	1	16.67	2	66.67	7
	Role-play	0	0	3	37.50	0	0	0	0	0	0	3
	Problem-solving technique	0	0	1	12.50	1	16.67	0	0	0	0	2
	Self-instruction	1	5.26	1	12.50	0	0	0	0	0	0	2
PDT	Emphasis on past experiences	3	15.78	1	12.50	3	50.00	2	33.33	3	100	12
	Focus on therapeutic relationship/alliance	3	15.78	0	0	4	66.67	1	16.67	2	66.67	10
	Interpretation	0	0	0	0	3	50.00	2	33.33	3	100	8
	Working with unpleasant emotions	1	5.26	0	0	3	50.00	1	16.67	3	100	8
	Transference	0	0	0	0	2	33.33	2	33.33	3	100	7
	Working with unconscious conflicts	0	0	0	0	1	16.67	1	16.67	3	100	5
	Therapeutic abstinence/neutrality	0	0	0	0	1	16.67	0	0	2	66.67	3
	Resistance analysis	0	0	0	0	0	0	0	0	3	100	3
	Therapist allows free selection of topic	1	5.26	1	12.50	0	0	0	0	1	33.33	3
*Psychotherapeutic techniques*												
General	Skills training	3	15.78	7	87.50	0	0	1	16.67	0	0	11
	Advice/support	1	5.26	3	37.50	5	83.33	0	0	1	33.33	10
	Empathy	2	10.52	0	0	5	83.33	1	16.67	1	33.33	9
	Emotional activation	0	0	0	0	3	50.00	0	0	2	66.67	5
	Focus on strength	2	10.52	1	12.5	2	33.33	0	0	0	0	5
	Relaxation	0	0	4	50.00	0	0	0	0	0	0	4

Notes: *n* = number of used techniques, % = percentage of used techniques, total = total number of included techniques, CBT = cognitive-behavioural techniques, PDT = psychodynamic techniques, and general = general psychotherapeutic techniques.

The label ‘cognition-focused treatment’ was chosen because nearly all treatments (94.7%) worked with dysfunctional cognitions. The activation-focused treatment included diverse behavioural activities, such as skills training (87.5%), homework assignments (87.5%), activation (75.0%), and relaxation (50.0%). The empathy-focused treatment clusters encompassed treatments that frequently utilised empathy (83.3%) and advice/support (83.3%). In the unspecific-focused treatment cluster, no pattern of any specific psychotherapeutic techniques was identified. Treatments from the psychodynamic-focused treatment cluster used typical psychodynamic techniques, such as emphasis on past experiences (100%), interpretation (100%), transference (100%), and working with unconscious conflicts (100%).

The results from the cluster analysis were validated through two additional analyses. First, a linear discriminant analysis was carried out. Four of the five clusters were confirmed. These confirmed clusters were: cognition-focused treatment, activation-focused treatment, empathy-focused treatment, and psychodynamic-focused treatment. Studies from the unconfirmed unspecific-focused treatment were classified as belonging to the cognition-focused cluster.

The second validation procedure used three random samples from 80% of the assigned studies. Using these three random samples, three hierarchical cluster analyses were carried out. Cohen's *κ*, as an agreement of the classification between the initial cluster and the new cluster, ranged from *κ* = .59 to *κ* = 1. The median *κ* = .77 was excellent. The *κ* values marginally differed between the clusters (cognition-focused treatment: range = .75–.87, Mdn = .87; activation-focused treatment: range = .67–1, Mdn = .71; empathy-focused treatment: range = .63–.79, Mdn = .76; unspecific-focused treatment: range = .59–.87, Mdn = .76; psychodynamic-focused treatment: range = .78–1, Mdn = .89).

### Temporal change in techniques used within approaches

Comparing the use of psychotherapeutic techniques in older (prior to 1995) and newer (after 1996) studies of the same approach did not reveal substantial changes. No significant differences were found for STPP treatments (all *p*s > .185). For CBT treatments, the only difference was an increase in the use of treatment goal setting in the newer studies (*p* = .032).

## Discussion

Our findings showed that psychodynamic techniques were exclusively used in treatments labelled as STPP, while cognitive-behavioural techniques were more commonly used in both treatment approaches. This result corresponds with other findings that demonstrate that STPP therapists apply a significant number of cognitive-behavioural techniques, in addition to psychodynamic strategies (Ablon & Jones, 1998; cited by Larsson, 2010). The hypothesis that the presence of psychotherapeutic techniques changes over time within the two approaches was not confirmed by our study. This result contrasts with previous findings that indicated that there was an increasing integration of strategies and techniques with different treatment approaches (Beitman et al., 1989; Goldfried, 2009) or within one treatment approach (Streeck, 2000).

The empirical clustering of treatment approaches according to psychotherapeutic techniques resulted in five clusters, which showed distinct patterns in the psychotherapeutic techniques used. CBT treatments were divided into two clusters, labelled as cognition-focused treatment and activation-focused treatment. This finding partially corresponds with the findings of Cuijpers, Van Straten, Andersson, and Van Oppen (2008), who divided CBT treatments into two types of intervention strategies. The first was labelled as a CBT approach, with an emphasis on cognitive restructuring (cognition-focused treatment in our study). The second approach used activation and cognitive restructuring, which corresponds to our activation-focused treatment. The STPP treatments were split into three clusters: empathy-focused treatment, unspecific-focused treatment, and psychodynamic-focused treatment. This result partially supports the findings of Trijsburg et al. (2004). In their study, psychodynamic treatments were divided into psychoanalytic interventions (i.e. transference, working through resistance), which corresponds to our psychodynamic-focused treatment, and into a psychodynamic intervention (i.e. to explore feelings and experiences at a more conscious level, and to address relationships outside the therapy), which corresponds to our empathy-focused treatment or the unspecific-focused treatment. The cluster of empathy-focused treatments in our study included techniques from STPP and CBT and, thus, can be regarded as a group of integrative approaches.

## Limitations

The accuracy of our investigation is restricted by the accuracy of the treatment descriptions in the publications. Thus, if the terms used in the descriptions were ambiguous, the coding techniques were difficult to use. For example, we found it challenging to code whether or not a term indicated ‘working with unpleasant emotions’ or ‘emotional activation’. Nevertheless, most codes were rated with good to excellent reliability.

Similarly, it is problematic to assume that a specific term always refers to a specific technique. It is possible that different authors used the same term to refer to what are actually different techniques. This limitation has already been discussed elsewhere and reflects the fact that a standardised vocabulary of psychotherapeutic techniques and other intervention components is lacking (Abraham & Michie, 2008). Consequently, future efforts should be made to develop a common language or set of terminology (Abraham & Michie, 2008; Beitman et al., 1989; Ryle, 1978).

Our study is based on an unbalanced selection of CBT and STPP trials (2:1). This might have affected the findings of the cluster analysis, since the techniques from CBT are coded more often. Unfortunately, trials with STPP are done in a limited number of eligible studies. In order to extend the database we included more CBT trials.

Many studies had short treatment descriptions, which we primarily classified as unspecific-focused treatment (*n* = 6). Thus, these treatment descriptions did not contribute substantial information to our findings. In similar future investigations, studies with short treatment descriptions should either be excluded or the treatment manuals should be consulted in order to gain better information about the treatments.

Some shortcomings of the rating manual need to be considered. First, the manual could be extended to include techniques used in the treatment of other psychological disorders (e.g. exposure in anxiety disorders). Problems with the classification of treatments are especially present if the general principle of change (i.e. exposure) overlaps with all of the specific treatments for posttraumatic stress disorder (Gerger et al., 2014). Additionally, some techniques could be removed because they are rarely mentioned in treatment descriptions (e.g. inclusion of significant others and self-disclosure). Finally, some definitions should be reworked in order to improve clarity (e.g. empathy).

## Conclusions

Our findings have important implications for meta-analyses of psychotherapy outcome studies. In meta-analyses, it is common practice to pragmatically categorise treatments according to the labels used in the primary studies. Our study suggests that this approach might be misleading since treatments with the same label might differ substantially in the techniques applied and, thus, differences between subgroups might be obscured. Therefore, it seems necessary to increase efforts to classify treatments with respect to the techniques used, similar to how it was done in a recent meta-analysis on the efficacy of psychotherapy for PTSD (Watts et al., 2013). A good description of the new types of interventions is especially important in cases of new treatment approaches, since in newly developed treatment effects are often initially overestimated (allegiance bias) (Munder, Brütsch, Leonhart, Gerger, & Barth, 2013; Munder, Flückiger, Gerger, Wampold, & Barth, 2012; Munder, Gerger, Trelle, & Barth, 2011), and explanations within the treatment descriptions are rare.

This investigation also found that CBT and STPP treatments in RCTs could be clearly differentiated by their use of techniques, as described in the papers. Thus, our results do not indicate a substantial overlap in techniques between these two major approaches. The assumption that treatments equalise over time in terms of the psychotherapeutic techniques used was not proven. However, a substantial number of STPP treatments from the empathy-focused treatments integrated CBT techniques, such as patient education and reinforcement. An adoption of STPP techniques in CBT was not found.

These findings support the notion that CBT is a unique therapeutic position, and even STPP therapists integrate CBT techniques. From the opposite perspective, the non-adoption of techniques from STPP, such as the focus on therapeutic relationships, could indicate potential for CBT to become more effective. Such a conclusion is based on evidence from process research that demonstrates a high impact of the therapeutic relationship on the treatment outcome (Flückiger, Del Re, Wampold, Symonds, & Horvath, 2012).

Extracting data regarding therapeutic techniques was feasible in this study. However, the description of the therapeutic approaches can be improved in order to make results of randomised studies more clinically meaningful. The importance of a common vocabulary is necessary (Abraham & Michie, 2008; Beitman et al., 1989; Ryle, 1978). Common terminology would allow for a better distinction between the different psychotherapeutic techniques that are used in interventions. Likewise, the extracting procedure for systematic reviews would be facilitated through this common terminology.

In the description of the studies, the applied intervention's techniques might be reported, in addition to the standardised labels. A common vocabulary should be based on the shared knowledge and understanding of therapists and researchers from different backgrounds in order to achieve a consensus in definitions and close the gap between research and practice (Goldfried, 2010). We hope that our study contributes to developing such definitions.

## Appendix 2. Rating manual for psychotherapeutic techniques, structured in cognitive-behavioural techniques, psychodynamic techniques and general psychotherapeutic techniques (version 3).

**Table:**

Label	Definition	Synonyms
*Cognitive-behavioural techniques*		
Reinforcement (positive/negative)	The therapist uses systematic reinforcement to establish new behaviours. The therapist encourages and rewards desirable and reasonable behaviour. Negative consequences may be systematically used to weaken disruptive behaviours, such as aggression or impulsivity	Reward, praise, (in-) direct punishment, deprive, aversive cue, elimination, positive cue, aversive conditioning, response cost
Role-play	The therapist develops a role-play situation with the patient and practices active role-play situations	Psychodrama, role reversal, role swap, change of roles, surrogate desensitisation
	*This label should not be rated at the same time as ‘skills training’*	
Problem-solving technique	The therapist uses a problem-solving technique: the process of problem identification, description, goal definition, generation of possible solutions, decision-making (weighing costs and benefits) and evaluation of new experiences	Problem identification, goal definition, generation of possible solutions/brainstorming, decision-making, evaluating of new experiences
	*This label should not be rated at the same time as ‘skills training’*	
Education about the disorder	The therapist gives information about the actual clinical problem; he teaches theoretical models explaining the origin and the possibilities for the modification of a specific disorder	Psycho-education, psycho-educative arrangements, teaching, information about the disorder
Education about the therapeutic strategy	The therapist describes the planned therapeutic strategy	Explaining a therapy rationale/model/technique, individual model of the disorder, behavioural analysis
Structuring of treatment/session	The therapist summarises key issues of the session, sets a session agenda and puts emphasis on certain topics during the session. The treatment consists of different phases	
Setting treatment goals	The therapist determines the goals of the therapy together with the patient	Aims, targets, purposes, objectives of therapy, discussion and formulation of partial stages for treatment goals
Label	Definition	Synonyms
Self-instruction	The therapist teaches the patient to control his behaviour through functional self-verbalisation	Internal state, self-verbalisation, verbal self-instructions, establishing guiding self-statements
Activation	The therapist explores pleasant activities with the patient and makes plans on how to materialise them	
Working with dysfunctional cognitions	The therapist uses techniques such as identification of maladaptive automatic thoughts and cognitive schemata and correction of thinking errors	Identification of irrational beliefs and negative thoughts, relationship of thoughts, feelings and behaviours, ABC technique, reframing, schemas
Homework assignments	The therapist assigns extratherapeutic tasks. The therapist talks with the patient about the homework from the last session	Homework, daily exercises, daily tasks, preparation for the next session, diary
	*This label should not be rated at the same time as ‘monitoring’ and ‘activation’*	
Monitoring	The patient observes his or her cognition, emotion and other behaviours. This technique is mainly thought as diagnostic procedure and not as therapy	Registration
	*This label should not be rated at the same time as ‘homework assignments’*	
*Psychodynamic techniques*		
Label	Definition	Synonyms
Therapist allows free selection of topic	The therapist allows the patient to say everything that comes to his mind	(Free-) association, connotation, space for (self-) exploration
Therapeutic abstinence/neutrality	The therapist shows consistent attention to the statements of the patient. He is equally responsive to all of the patient's behaviours, wishes and feelings	Impartial, unbiased, non-judgmental, value-free, neutral attitude/stance
	*Code 0 if the therapist is active and engaged*	
Focus on therapeutic relationship/alliance	The therapist monitors the patient's view of the therapeutic relationship and progress of therapy	Discussion about the therapeutic relationship, fostering the therapeutic relationship, collaboration, alliance ruptures
	*This label should not be rated at the same time as ‘transference’*	
Label	Definition	Synonyms
Transference	The therapist reflects on the patient's reactions towards him and uses this material to work with the patient on his relationship patterns	
	*This label should not be rated at the same time as ‘therapeutic relationship’*	
Resistance-analysis	The therapist draws the patient's attention to his resistance to insight/progress in treatment and potential reasons	Opposition, patient refuses suggestions
Working with unconscious conflicts/relational patterns	The therapist discusses the patient's unconscious conflicts between wishes, needs and fears as reasons for the clinical problem	Confrontation with unconscious conflicts/relational patterns/clarification
	*This label should be rated at the same time as ‘interpretation’*	
Interpretation	The therapist makes something previously unconscious for the patient conscious, by interpreting feelings, thoughts and behaviours	Insight enhancing, interpretative utterances, interpret subconscious issues and identify recurring patterns, insight (*insight should be rated at the same time as ‘working with unconscious conflicts’*)
Working with unpleasant emotions	The therapist focuses on unpleasant emotions and encourages patients to experience them	Working on feelings of guilt, anger, hate, fear, jealousy, shame
Emphasis on past experiences	The therapist focuses on events and significant others of the patients’ past. He links the patient's current feelings and perceptions to his biography	Childhood experiences, past events, link the past with the present
*General psychotherapeutic techniques*		
Label	Definition	Synonyms
Self-disclosure	The therapist reports on personal experiences	Self-revelation, the therapist talks about himself
Skills training	The therapist utilises skills training, by teaching specific verbal and nonverbal behaviours and by encouraging the patient to practice these behaviours	Ability, capability, awareness, mindfulness, assertiveness training, time-management, stress-management
	*This label should not be rated at the same time as ‘role-play’ or ‘problem-solving technique’*	
Emotional activation	The therapist activates the patient's emotional responsiveness towards different experiences	Experiencing, encouraging feelings, facilitating feelings
Label	Definition	Synonyms
Empathy	The therapist uses empathic responding to help the patient to connect with his feelings, values and wishes	
Inclusion of significant others	The therapist includes in the therapy other people, who are important to the patient	Involvement of partner, family, friend(s)
Advice/support	The therapist suggests what to do in a particular real life situation	Tip, advice, support, aid, encouragement, assistance, help
Relaxation	The therapist uses techniques which reduce physiological arousal	PMR, imagination, autogenic training
Focus on strength	The therapist focuses on patient's resources (social network, competencies, self-efficacy, etc.).	Resource activation

## Appendix 3. Cohen's kappa of each category.

**Table:**

Technique	Cohen's kappa
Reinforcement (+/−)	.717
Role-play	.481
Problem-solving technique	.481
Education about the disorder	1.00
Education about the therapeutic strategy	.919
Structuring of treatment/session	.815
Setting treatment goals	.730
Self-instruction	.376
Activation	.869
Working with dysfunctional cognition	.830
Homework assignments	.879
Monitoring	.900
Therapist allows free selection of topic	1.00
Therapeutic abstinence/neutrality	.790
Focus on therapeutic relationship/alliance	1.00
Transference	.919
Resistance-analysis	.847
Working with unconscious conflicts/relational patterns	.898
Interpretation	.919
Working with unpleasant emotions	.655
Emphasis on past experiences	.940
Self-disclosure	1.00
Skills Training	.826
Emotional activation	.696
Empathy	.508
Inclusion of significant others	1.00
Advice/support	.767
Relaxation	1.00
Focus on strength	.730
